# How to keep registered nurses working in New Zealand even as economic conditions improve

**DOI:** 10.1186/s12960-018-0312-x

**Published:** 2018-09-10

**Authors:** Willoughby Moloney, Des Gorman, Matthew Parsons, Gordon Cheung

**Affiliations:** 1 School of Nursing, University of Auckland, 85 Park Road, Grafton, Auckland, New Zealand; 2 Health Workforce New Zealand, Wellington, New Zealand; 3 Faculty of Medical and Health Sciences, University of Auckland, Auckland, New Zealand; 4 Business School, University of Auckland, Auckland, New Zealand

**Keywords:** Nursing turnover, Nurse retention, Work engagement, Burnout, Workload, Work-life interference

## Abstract

**Background:**

Many registered nurses (RNs) increased their participation in the New Zealand health workforce during the Global Financial Crisis (GFC), resulting in low vacancy rates. However, based on the documented impact of improving economies, a mean RN age of about 50, and just-agreed substantive increases in RN pay rates, it is likely that many will soon leave or reduce the hours they work. This study aims to investigate whether improved financial security will encourage RNs to leave or reduce their work commitment and to identify the factors that influence such intentions.

**Methods:**

An exploratory study using a cross-sectional survey design. Data were collected in 2014-2015 via an e-survey of 2,910 RNs in New Zealand. Data were analysed by regression.

**Results:**

We found that due to “burnout” and low “work engagement”, both of which are strongly affected by workload and work-life interference, 22.6% of the RNs surveyed plan to leave work altogether and a further 32% plan to reduce their workforce participation when their financial situations improve.

**Conclusions:**

The findings justify the urgent cooperative development, implementation and evaluation of a comprehensive suite of RN ‘retention’ measures involving national nursing organisations, the RN regulator and health system employers and funders.

## Background

Given that RNs make up the largest professional group in healthcare, nursing shortages are an important policy concern. Such shortages have negative impacts on health costs, patient care and staff morale [1–6]. Relatively low RN staffing levels are associated with negative health outcomes such as increased mortality rates [7, 8], increased patient medical complications [8] and reduced nursing efficiency and safety [4].

Since the Global Financial Crisis (GFC) began in 2007, the number of doctors and registered nurses (RNs) in paid employment has increased [9, 10]. There is concern, however, that those practitioners who re-entered or extended their involvement in the workforce during the GFC may leave their jobs or reduce their hours once their financial situation improves [9, 11]. In New Zealand, as elsewhere, this will be concurrent with an ageing population, which has a greater need for healthcare [12], increasing levels of multi-morbidity [13], and an ageing healthcare workforce that is nearing retirement [10, 14]. The Nursing Council of New Zealand workforce survey database shows a stable workforce in regard to the RN to population ratio over the last 5 years (11.03/1 000 in 2012 and 11.1/1 000 in 2017) [15]. In regard to RNs in direct care roles (as defined by the OECD) [15], most recent data show a ratio of 10.6/1 000 in New Zealand, compared to 7.99 in the United Kingdom, 9.9 in Canada and 11.7 in Australia. The United States of America does not publish direct care role data, but in relation to the total RN workforce, there were 11.3/1 000 population at the most recent US census, compared to 11.1 in New Zealand.

To achieve parity with the Australian ratio, and consequently to achieve usual compliance with the ‘safe staffing levels’ adopted in New Zealand [16], our modelling of the Council data indicate that a 11% increase in the RN workforce is necessary. Current entry levels into the RN workforce (3 261 new entrants and 962 re-entrants per annum) will maintain current RN to population ratios if current exit rates and average full-time equivalent workloads are maintained, whereas an additional 510 entrants or re-entrants will be required each year over the next decade to raise the RN to population by 10%. In contrast to this assumption of stable RN exit rates from the workforce, the Council data show a steady decline in the retention of RNs from the age of 50 years [17] and it is predicted that over 50% of the present RN workforce will retire by 2035 [18]. The combination of increasing demand for healthcare, an ageing nursing workforce in the context of an age-dependent exit rate from the profession, and an increase in nursing pay rates that has just been agreed by the national RN organisation and the New Zealand Government, which will improve household finances, leads to a forecast of an imminent severe RN shortage.

A strong correlation has been reported between job dissatisfaction and nursing turnover [19, 20] and the constructs of satisfaction and employment alternatives underpin most traditional concepts of employee turnover. The literature on turnover often focuses on career orientation and occupational commitment, where employees view their job as a line of work that they wish to pursue for many years [21]. These employees are likely to display higher work engagement [22] and lower intention to leave [23]. In support of this, Moore [24] found that a sense of professionalism mitigated RN desire to leave the profession despite the impact of burnout, while Angerami et al. [25] determined that RN motivation to remain in the profession related to an attachment to nursing, even though their work was ‘not recognised and they were poorly paid’.

Previous research shows that RNs may reduce their participation in the workforce due to accumulated burnout precipitated by heavy workloads [26–29]. This is because high workloads can result in increased levels of stress when the situation requires significant effort to sustain an expected performance level [30]. Further, the increase in dual career relationships, where both partners are working outside the home, has made balancing work and home responsibilities such as caring for children more challenging [31]. This increases the need for a harmonious balance between work-life and home-life. Women represent 91% of the nursing workforce in New Zealand, many of whom have family responsibilities [32]. Many RNs report that work adversely interferes with their home life [33]. Conflict between work and family has been shown to lead to burnout and profound long-term consequences for workers [29, 34].

To date, there has been little examination of how financial factors affect transitions into and out of the RN workforce. We do know that, during a recession, RNs who have not been working or who have been working part-time may re-join the workforce or change to full-time status because their partners have a reduced income, so as to maintain their household economic security [11, 35]. Some RNs may move to holding more than one job, while others who were working in different sectors may return to nursing if their current occupational positions become tenuous. However, research is needed into what happens when economic circumstances improve, as they have since the end of the GFC and will do so in a series of new pay rate steps. The research question then is primarily to investigate whether improved financial security will encourage RNs to leave or reduce their work commitment and, secondarily, to identify the factors that influence such intentions.

The results will help to guide and establish priorities for future research that can inform policy initiatives to improve the retention of RNs and reduce the potential for a severe nursing shortage. The systemic long-term ambition is greater RN work satisfaction and status, RNs working to their full scope of practice, a consequently stable and productive workforce, and better healthcare efficiency.

## Method

The exploratory study used a cross-sectional survey design. Data on the experiences at work were collected via an e-survey in 2014/2015 from 2 175 RNs working in New Zealand. Firstly, the experiences at work of the RNs who intended to leave the profession when their financial situations improve and those of the RNs who intended to stay when their financial situations improve were compared with independent samples *t* tests. Then the experiences at work of the RNs who intended to reduce work hours when their financial situations improve and those who intended to maintain their work hours when their financial situations improve were also compared. Due to multiple comparisons (20 in each analysis) being conducted, Bonferroni adjustment was adopted to control for the overall type I error rates, such that the type I error rates (alpha level) of 0.00 25 and 0.00 05 were used to conclude statistical significance in the *t* tests. These type I error rates will give overall type I error rates of 0.05 and 0.01. Finally, regression analysis was used to test which factors (identified by the literature as having an effect on RN turnover) have the most significant effect on RN intention to leave the profession (in general condition without assumption of financial improvement). The correlation coefficients among the independent variables ranged from − 0.60 9 to 0.63 6, with 90% of the correlation coefficients lie with − 0.37 and 0.45. Hence, multicollinearity should not be a concern in the regression analysis. Besides, relative weight analysis [36] was conducted to supplement the regression analysis to examine the unique contribution of each independent variable in explaining the variance of intention to leave the profession.

## Results

The sample was made up of 93.9% women and 6.1% men. Ages ranged from 18 to 75, with a mean age of 48.8 (s.d. = 11.54) years. Some 12.3% were working in primary healthcare, while the remainder worked in a range of secondary and tertiary settings. Years of tenure in current role ranged from less than one to 45, with a mean tenure of 7.1 (s.d. = 6.97) years. Highest reported reasons for working part-time were better work-life balance (32.1%), to care for dependent children (30.3%) and to phase in retirement (6.5%).

Approximately 15% of RNs had returned to the profession and 36% had increased their hours of work in the last 5 years due to financial need. Among those who had returned to RN employment for financial need, 25.8% intend to leave again as financial circumstances improve (among those RNs who had not returned to the profession in the last 5 years due to financial need, 22.1% intend to leave). This indicates a potential loss of 22.6% of the nursing workforce. Of those who had increased their hours, 65% intend to reduce their hours when their finances improve (among those who had not increased their hours of work in the last 5 years due to financial need, 44% intend to reduce their hours). When combined, these figures show that almost 55% of RNs plan to reduce their participation in the workforce to some degree.

Table 1 compares the RNs who want to stay and the RNs who intend to leave the profession if their financial situations improve. Those who intend to leave the profession are significantly older and report greater burnout, lower work engagement, lower career orientation, higher workload, greater work-life interference and less access to flexible hours. They also experience less supervisor support, less colleague support and less organisational support. They do not feel as safe from workplace bullying, perceive lower autonomy in their role and have personal values less congruent with their employer’s values and lower self-efficacy at work. Finally, they are less satisfied with their jobs and pay.

**Table 1:** Comparisons of RNs with intentions to stay/leave as finances improve

Variables	RNs who will stay even if finances improve	RNs who will leave as finances improve
Count (%)	1 946 (66.9%)	658 (22.6%)
Gender (%)		
Female	94	94
Male	6	6
	Mean (s.d.)	Mean (s.d.)
Age*	47.45 (11.48)	52.02 (11.09)
No. of dependent children	0.72 (1.17)	0.55 (1.03)
Percentage of contribution to household income	64.34 (27.57)	65.09 (27.93)
High burnout* (*1 = low to 7 = high*)	3.18 (1.09)	3.77 (1.22)
High work engagement* (*1 = low to 7 = high*)	4.83 (0.90)	4.34 (0.90)
Consider nursing as a long-term career* (*1 = low to 5 = high*)	4.05 (0.90)	3.62 (1.02)
High workload (pressure/urgency at work)* (*1 = low to 5 = high*)	3.24 (0.60)	3.39 (0.61)
Work impacts negatively on personal life (work-life interference)* (*1 = low to 5 = high*)	2.95 (0.90)	3.29 (0.94)
Access to flexible hours* ***(****1 = low to 5 = high*)	2.19 (1.24)	1.98 (1.22)
Good supervisor support* (*1 = low to 5 = high*)	3.17 (0.97)	2.90 (0.99)
Good colleague support* (*1 = low to 5 = high*)	3.64 (0.70)	3.51 (0.74)
Good organisational support* (*1 = low to 7 = high*)	3.57 (1.66)	3.10 (1.56)
Feel safe from workplace bullying* (*1 = low to 5 = high*)	3.61 (0.81)	3.33 (0.87)
Have access to professional development (*1 = low to 7 = high*)	5.09 (1.01)	4.98 (1.09)
Satisfied with pay* (*1 = low to 7 = high*)	3.85 (1.35)	3.56 (1.38)
Have autonomy in their role* (*1 = low to 7 = high*)	5.45 (1.24)	5.21 (1.28)
Feel personal values align with employer’s values* (*1 = low to 7 = high*)	4.51 (1.49)	4.10 (1.61)
Have self-efficacy/confidence at work* (*1 = low to 7 = high*)	5.30 (0.91)	5.02 (0.94)
Job satisfaction* (*1 = low to 10 = high*)	6.96 (2.02)	5.93 (2.26)

Variables with * are statistically significantly different across groups (*p* < .00 1)

Table 2 compares the RNs who want to maintain hours and the RNs who intend to reduce hours if their financial situation improves. Here there is no significant difference in age, but those who intend to reduce hours contribute somewhat more to their family income. They report greater burnout, lower work engagement, lower career orientation, higher workload, greater work-life interference and less access to flexible hours. They experience less supervisor support, less colleague support and less organisational support. They do not feel as safe from bullying, perceive lower autonomy in their role, have personal values less congruent with employers’ values, have lower self-efficacy at work and are less satisfied with their jobs and pay.

**Table 2:** Comparisons of RNs with intentions to maintain/reduce hours as finances improve

Variables	RNs who will maintain hours even if finances improve	RNs who will reduce hours as finances improve
Count (%)	1 131 (38.9%)	1 492 (51.3%)
Gender (%)		
Female	94	94
Male	6	6
	Mean (s.d.)	Mean (s.d.)
Age	49.04 (11.51)	48.31 (11.65)
No. of dependent children	0.73 (1.19)	0.62 (1.09)
Percentage of contribution to household income*	60.67 (28.10)	67.96 (26.57)
High burnout* (*1 = low to 7 = high*)	3.02 (1.09)	3.61 (1.14)
High work engagement* (*1 = low to 7 = high*)	4.92 (0.89)	4.52 (0.90)
Consider nursing as a long-term career* (*1 = low to 5 = high*)	4.07 (0.91)	3.81 (0.97)
High workload (pressure/urgency at work)* (*1 = low to 5 = high*)	3.17 (0.61)	3.36 (0.60)
Work impacts negatively on personal life (work-life interference)* (*1 = low to 5 = high*)	2.81 (0.89)	3.25 (0.92)
Access to flexible hours* (*1 = low to 5 = high*)	2.29 (1.24)	2.00 (1.24)
Good supervisor support* (*1 = low to 5 = high*)	3.21 (0.98)	3.01 (0.98)
Good colleague support* (*1 = low to 5 = high*)	3.66 (0.71)	3.57 (0.71)
Good organisational support* (*1 = low to 7 = high*)	3.74 (1.68)	3.22 (1.61)
Feel safe from workplace bullying* (*1 = low to 5 = high*)	3.64 (0.81)	3.42 (0.86)
Have access to professional development (*1 = low to 7 = high*)	5.14 (1.01)	5.01 (1.03)
Satisfied with pay* (*1 = low to 7 = high*)	3.99 (1.36)	3.60 (1.34)
Have autonomy in their role* (*1 = low to 7 = high*)	5.53 (1.24)	5.29 (1.25)
Feel personal values align with employer’s values* (*1 = low to 7 = high*)	4.68 (1.49)	4.22 (1.53)
Have self-efficacy/confidence at work* (*1 = low to 7 = high*)	5.39 (0.91)	5.11 (0.92)
Job satisfaction* (*1 = low to 10 = high*)	7.17 (2.03)	6.28 (2.15)

Variables with * are statistically significantly different across groups (*p* < .00 1)

Table 3 shows results of the multiple regression model, which explains 14% of the variance in intention to leave the profession. Those variables shown to have explained more than 1% of the variance of the intention to leave the profession (relative weights larger than 0.01) are career orientation (2.8%), job satisfaction (3.2%), burnout (1.6%) and work engagement (2.0%). In other words, greater intention to leave nursing is predicted by a lower career orientation, lower job satisfaction, greater burnout and lower work engagement.

**Table 3:** Factors predicting RNs’ intention to leave profession

Independent variables	Unstandardised Coefficients	Standardised Coefficients
	B (Std. error)	Beta
(Constant)	3.82 5 (0.27 8)	
Age	− 0.00 3 (0.00 2)	− 0.04 3
No. of dependent children younger than 14	− 0.02 4 (0.02 1)	− 0.02 6
No. of dependent adults	0.09 8 (0.04 7)	0.04 2
Contribution to household income	− 0.00 1 (0.00 1)	− 0.03 2
Health (*1 = low to 5 = high*)	0.03 4 (0.01 9)	0.03 9
Tenure (*years*)	0.00 3 (0.00 3)	0.02 3
Job satisfaction (*1 = low to 10 = high*)	− 0.07 6* (0.01 2)	− 0.19 1
Consider nursing as a long-term career (*1 = low to 5 = high*)	− 0.12 0* (0.02 1)	− 0.13 6
High work engagement (*1 = low to 7 = high*)	− 0.08 6* (0.02 8)	− 0.09 4
High burnout (*1 = low to 7 = high*)	0.06 6* (0.02 4)	0.09 1
High workload (pressure/urgency at work) (*1 = low to 5 = high*)	0.03 1 (0.03 7)	0.02 2
Image of nursing in society (*1 = low to 7 = high*)	0.02 5 (0.01 6)	0.03 5
Emotional demand (hindrance) (*1 = low to 5 = high*)	− 0.00 7 (0.02 6)	− 0.00 6
Emotional demand (challenge) (*1 = low to 5 = high*)	0.05 0 (0.03 0)	0.03 9
Work impacts negatively on personal life (work-life interference) (*1 = low to 5 = high*)	0.02 1 (0.02 5)	0.02 3
Good supervisor support (*1 = low to 5 = high*)	0.05 1 (0.02 1)	0.06 0
Good colleague support (*1 = low to 5 = high*)	0.04 2 (0.02 6)	0.03 5
Good organisational support (*1 = low to 7 = high*)	0.01 6 (0.01 5)	0.03 1
Feel safe from workplace bullying (*1 = low to 5 = high*)	0.01 1 (0.02 8)	0.01 1
Have access to professional development (*1 = low to 7 = high*)	− 0.02 1 (0.01 8)	− 0.02 6
Satisfied with pay (*1 = low to 7 = high*)	0.03 0 (0.01 4)	0.04 8
Have autonomy in their role (*1 = low to 7 = high*)	0.01 1 (0.01 6)	0.01 6
Feel personal values align with employer’s values (*1 = low to 7 = high*)	0.01 6 (0.01 6)	0.02 8
Have self-efficacy/confidence at work (*1 = low to 7 = high*)	− 0.03 1 (0.02 4)	− 0.03 4
*R*^2^ = 0.13 9 (*F* = 14.46 9, *p* < 0.00 1)		

*N* = 2 175. Regression coefficients with * are statistically significant (*p* < .01)

## Discussion

This study shows that a quarter (22.6%) of the surveyed RNs plan to leave the workforce as their financial circumstances improve. If extrapolated to New Zealand’s nursing population, this would represent a loss of approximately 12 500 RNs. This will occur at the same time that demand-side pressures on health services increase due to an ‘ageing’ population and an increasing prevalence of multi-morbidity, and as supply-side stressors increase for a nursing workforce in which many are near retirement. A reduction in RN participation of this magnitude will result in greater workloads for those remaining, which has been shown to be one of the most significant reasons RNs choose to reduce their hours or leave the profession [27–29]. Therefore, the study, considered in concert with the predictable impact of the just-agreed increases in pay rates for RNs in New Zealand [5, 7], suggests the predicted fall off in nursing workforce availability will be greater and occur sooner than expected.

This study identifies important differences between those RNs who want to leave nursing and those who intend to stay when their financial situations improve and, similarly, between those who wish to maintain their hours and those who would reduce them. As might be expected, those intending to stay or maintain their working hours report greater job satisfaction, lower burnout and higher work engagement. As the regression confirms, their work attitudes are more positive and they show a greater long-term commitment to nursing as a career. This indicates that a focus on reducing burnout and improving work engagement is vital to retaining RNs.

The more positive attitudes of the stayers/maintainers are associated with a range of ways in which their jobs are of higher quality. They perceive greater autonomy and better support from colleagues, supervisors and their organisation, and experience lower levels of workload, work-life interference and bullying. It is interesting to note that in neither case does access to professional development differentiate the two groups, suggesting that this factor is less important in RN retention that the quality of the work itself, including its intrinsic satisfactions and strains.

Two key ways to reduce burnout and improve work engagement will be to reduce workload and work-life interference. Intense and demanding workloads have been shown to cause RNs to feel emotionally and physically exhausted, and to be unhappy about their ability to provide a desirable quality of care [37]. Furthermore, many RNs have been found to only work part-time, or casual hours, due to the need for work-life balance [38]. With the increase in dual-career relationships, the issue of work-life balance and flexibility has become more important. For many older RNs, an increasing need for work-life balance may be related to ‘slowing down’ prior to retirement. They may believe that they can extend their ‘working lives’ if they can integrate their work into a lifestyle that allows them real flexibility and more personal time.

Finding ways to delay the retirement of RNs is one of the necessary responses to any nursing shortage [39], such that more flexibility regarding work hours is essential. By contrast, more than half the RNs surveyed said they did not have access to flexible start and finish times, and a similar number also reported that they felt too tired after work to pursue personal interests and spend time with their family and friends. Clearly, work-life interference is a significant risk to the retention of RNs in the workforce.

It is important to consider whether extrinsic motivations to participate in nursing, such as economic need, have an impact on the quality of care delivered. There is little research on this subject; this study suggests that this is overdue. It would be imprudent to rely on RNs’ predominant altruistic culture and intrinsic motivation for holistic patient care to balance any negative effects arising from economic and other stressors and make RN work both meaningful and rewarding [40–43].

Efforts to keep those RNs who returned for financial need are essential given imminent severe workforce shortages. However, the results of this study, and others, show RNs with intrinsic motivations, such as a sense of self-efficacy, report lower intentions to leave [44]. It follows that training institutions and employers should focus on recruiting RNs who display intrinsic motivations to participate in nursing, in the expectation that they will become a more stable workforce that is less susceptible to economic fluctuations. The counter-argument is that many RNs who start out with intrinsic motivations might become disillusioned as they experience burnout in practice. It is essential therefore that regardless of recruitment practice, employers develop strategies to reduce burnout as recommended by this study.

A limitation of the study arises from the cross-sectional design. To minimise the potential for common-method bias, we used measures with well-established construct validity and internal reliability, and the questionnaire was structured to separate the predictors and criterion variables and included reverse-coded items. Nevertheless, our results cannot provide definitive information about cause-and-effect relationships. Furthermore, the study identifies RNs who intend to leave; however, it does not follow up these same RNs to discover whether their intentions were realised. Further longitudinal study designs are recommended.

### Implications for policy

A multi-faceted approach will be required to maintain and retain the nursing workforce. Three potential over-arching strategies are discussed, including increasing supply, reducing workload and work-life interference through the development of a comprehensive suite of RN ‘retention’ measures, and adapting the RN role. The first strategy is to increase the production of RNs by increasing nursing school intakes and to increase the recruitment of RNs from other countries. However, a substantive short-term impact of lifting RN production and recruitment is unlikely for the following reasons: the training of new RNs takes time and is expensive; there are no reliable tools for identifying candidates for RN training who are intrinsically motivated to participate in nursing; the supply of overseas trained RNs is unpredictable and subject to competition from other health systems that also rely on international recruitment to meet supply needs [45]; overseas trained RNs can find it difficult to adapt to the cultures of their patients and the highest productivity losses are incurred when ‘departing’ RNs are replaced with new graduates and those from overseas [1].

The second strategy is the development of a comprehensive suite of RN ‘retention’ measures. A process of cooperative development, implementation and evaluation is essential and must involve nursing organisations, the RN regulator, RN employers and health-funders. This needs to take into account the probable perverse impact of the just agreed RN pay rate increases, given that 51.3% of the RNs surveyed in the study indicated that they intended to reduce their work commitments as soon as their financial situations allowed. In addition to a review of nursing student selection processes and undergraduate pedagogy that addresses health need and consequently showcases desirable RN vocations, the suite will need to include the following: the ring-fencing of enough suitable ‘first-year’ RN jobs for mutually determined graduate numbers, and support that is appropriate for such an ‘internship’; an alignment of RN careers, training and professional development; an up-skilling of the leadership and followership capabilities of the RN workforce; a commitment to consistently meet agreed ‘safe’ staffing levels and the implementation of genuinely flexible and adaptive work rosters; and a values- and futures-based review of how RNs are deployed and employed [46].

A third strategy is the possible role and task transfer and substitution, as suggested by the latter ambition of the ‘retention’ suite, within the health workforce, and especially in regard to shifting the locus of control to citizens, remains problematic. In contrast to substantive role transfer when job descriptions are constructed on the basis of skills and knowledge as compared to profession-defined scopes of practice [47], or when consumer choice determines the model of care [48], our experience in workforce transformation is that a profession-based de-construction approach inevitably maintains roles that could be surrendered and those that are ‘given up’ often become stigmatised. We argue that a skills-matrix approach is most likely to result in the level of work-satisfaction necessary to underpin a stable, happy and productive RN workforce. [49, 50]. However, significant barriers will need to be overcome for such an approach to be implemented [50, 51]. The challenges include making a shift from provider-centric to consumer-centric models of healthcare; improving contextual and values-based leadership training as a fundamental domain of professionalism for all health professionals; developing less restrictive business models and funding systems; changing regulatory practice to facilitate innovation; and by not allowing the territorial behaviour of health professions to compromise efficient healthcare.

## Conclusions

Although the retention of all RNs is a concern, this study shows that those RNs who increased their work participation during the GFC are at greater risk of reducing their involvement in nursing as their household finances improve. This is imminent in New Zealand given just-agreed increases in RN pay rates. Serious shortcomings in healthcare would arise if 55% of RNs go ahead with the intentions they reported to us and reduce their workplace participation. We consequently recommend that a range of measures are urgently instituted, the most productive of which is likely to be a suite of retention measures developed and delivered by nursing organisations, the RN regulator, RN employers and health-funders. This study highlights that such initiatives must address burnout and work engagement by way of optimising workload and work-life balance.

## Abbreviations

GFC: Global Financial Crisis
OECD: Organisation for Economic Co-operation and Development
RN: Registered nurse
